# The effect of female circumcision on maternal and neonatal outcomes after childbirth: a cohort study

**DOI:** 10.1186/s12884-022-05316-4

**Published:** 2023-01-20

**Authors:** Soheila Rabiepour, Zeynab Ahmadi

**Affiliations:** 1 Professor Reproductive research center, Urmia university of Medical ScienceUrmia, Urmia, Iran; 2 Consultation on midwifery, Reproductive Health Research Center, Clinical Research Institute, Urmia University of Medical Sciences, Urmia, Iran

**Keywords:** Female circumcision, Maternal outcomes, Neonatal outcomes, Cohort study, Women, Delivery

## Abstract

**Background:**

Circumcision has many side effects and complications on women’s lives and affects their physical, mental, and social health. The present study aimed to investigate the effect of female circumcision on maternal and neonatal outcomes.

**Methods:**

Methods: A prospective cohort study was performed with 320 pregnant women by convenience sampling (160 individuals were circumcised and 160 were uncircumcised). Circumcision and its level were confirmed using observation. The data relating to demographic, midwifery history, medical history, maternal and neonatal outcomes were compiled using a questionnaire. All Statistical analyses were conducted by SPSS ver. 16.0. *P*-values less than 0.05 were considered significant.

**Results:**

The mean age of circumcised women was significantly higher vs. Uncircumcised women (28.92±6.2 vs. 25.42±4.8; *P* < 0.001). Circumcision was significantly higher in rural compared to urban areas (51.9% vs.18.1%). The level of female education, his parents, spouse, and husband’s employment status were significantly associated with circumcision (*P* < 0.001). 94.4% of uncircumcised nulliparous women and 86.9% of circumcised women experienced Intended Pregnancy (*P*=0.02). Eighty- five percent of women were circumcision type I. The higher mean duration of the second stage of labor, Second and Third degree of tear, and need for oxytocin in induction were significantly higher among circumcised women (*P* = 0.03, 0.003, 0.002, respectively).

The existence one stage of labor, Second and Third degree of tear, and the need for oxytocin in induction were significantly higher among circumcised women (*P* = 0.03, 0.003, 0.002, respectively).

**Conclusion:**

These findings underscore that Circumcision a prevalent predictor of poor neonatal outcomes and delivery processes, therefore this circumcised women needs intensive care during delivery.

## Background

Female circumcision as a traditional and non-therapeutic surgery [1] involves the removal or damage to some part or the whole external genitalia of women without any medical reason [2]. This world health problem is considered violence against women’s reproductive rights [1].

Circumcision is a harmful problem rooted in the cultural background and incorrect religious beliefs of individuals [3] and is usually conducted by elderly women, traditional midwives, and even hairdressers in non-sterile conditions by tools such as razors, knives, and broken glass without any anesthesia [4].

The outbreak of circumcision varies around the world, and reportedly thefemale circumcision occurs throughout the world but it is more prevalent in the west, east and northeast Africa, some Asian and Middle Eastern countries, and some immigrant communities in Northern America and Europe [5, 6]. Worldwide 200 million women have undergone female genital circumcision, and more than 3 million girls are at risk every year [7]. Although surveys conducted across Africa, the Middle East, and Southeast Asia document FGM prevalence rates ranging from 1% (Uganda, Cameroon) to more than 95% (Guinea and Somalia) [7]. The outbreak of circumcision in Iran has been reported between 20 and 70% in different studies and regions [8–10].

There are four levels of circumcision according to the World Health Organization classification including the first level: removal of the whole or part of the clitoris or prepuce, level 2: removal of the whole or part of the clitoris and minor labia with or without cutting the labia major, level three: removal of part or the whole external genitalia and suturing and tightening of the vaginal entrance; level 4, which is any other dangerous operation on the female’s vagina for nonmedical purposes, such as piercing, needling, splitting, etc., and no tissue is removed at this type of circumcision [11].

The World Health Organization has highlighted the abolition of female genital circumcision. The Great Britain has illegalized the circumcision since 1985 [12]. According to research by the World Health Organization, various reasons, including better health, increasing girls’ development and their preparation for marriage, controlling women’s sexual desires before marriage, girl’s resistance to illicit sexual acts, purity and beauty of women and there is the process of keeping the past generations’ customs for circumcision [2, 13].

Females’ circumcision can cause physical, mental and psychosocial health problems, including both short-term complications of the procedure and long-term complications. The latter problems range from urinary to sexual issues and problems related to childbirth [5, 14–16]. The effects of circumcision on maternal and neonatal outcomes have been investigated in several studies, including postpartum hemorrhages, perineal tears, rectovaginal fistula, increased risk of cesarean section, prolonged labor and prenatal resuscitation and premature neonatal death, stillbirth and hospitalization [1, 17–21].

However, the extent and severity of maternal and neonatal outcomes in circumcised individuals are not different from uncircumcised ones [12]. However, the extent and severity of maternal and neonatal outcomes in circumcised individuals are not different from uncircumcised ones [12]. In contrast, postpartum hemorrhage, emergency cesarean section, and third-degree vaginal tears were associated with circumcision [18, 19], prolonged duration of the second stage of labor, difficult delivery, higher death rates, lower Apgar scores were reported higher among circumcised women [17, 18]. The World Health Organization estimates that about 10–20 babies’ death per 1000 childbirth is due to circumcision [13].

Despite global efforts to prevent females’ circumcision, there is a knowledge gap regarding evidence-based care to improve the health outcomes in circumcised women, given the lack of denial of the issue and insufficient evidences on the level of female’s circumcision and probable consequences of pregnancy and delivery in Iran. The aim of this study was to determine the maternal and neonatal outcome in circumcised women referring to the delivery ward of Imam Khomeini Hospital in Piranshahr,, a city located in West Azarbaijan Province - northwest Iran..

## Methods

### Type of study and aim

We performed a prospective cohort study of pregnant women between 2019 to 2021 in the delivery ward of Piranshahr Imam Khomeini hospital. In order to investigate the effect of female circumcision on neonatal and neonatal outcomes we compared neonatal *matenal* and neonatal-related factors between women with and without genital circumcision.

### Ethics approval and consent to participate

All methods were carried out in accordance with relevant guidelines and regulations and all experimental protocols were approved by Urmia University of Medical Sciences/Iran licensing committee and the ethical code is (IR.UMSU.REC.1398.080). In this study informed consent was obtained verbally from all subjects with the favorable opinion of the ethics committee(the Licensing Committee of Urmia University of Medical Sciences/Iran with the code of ethics(IR.UMSU.REC.1398.080). Since the illiterate participants were also involved in this study, informed consent was obtained from the legal guardians of the illiterate participants.

All methods were carried out in accordance with relevant guidelines and regulations and all experimental protocols were approved by the Urmia University of Medical Sciences/Iran licensing committee the ethical code is (IR.UMSU.REC.1398.080). In this study, informed consent was obtained verbally from all subjects with the favorable opinion of the ethics committee.

### Sample size and sampling method

A total of 320 pregnant women were evaluated through convenience sampling. Assume the rate of emergency is 2.7 and 3.18% in the two groups with and without circumcision [19] at α = 0.05, the required minimum sample size for having an 80% power (i.e., β = 0.2), 156(≈160) members was specified in each group.

### Inclusion and exclusion conditions

Inclusion criteria included single pregnancy and people who were not selected for elective cesarean section.

### Data collection method

The data collection method included observation (observation of the genital tract in terms of confirmation of whether or not the circumcision was performed and its level) and a questionnaire containing 4 sections including demographic questions and questions on circumcision, history of pregnancy, medical history, and obstetric consequences. At the time of admission, in the delivery ward at the beginning of the project, a genital examination was performed in terms of confirmation of circumcision performance and the level of genital examination circumcision, then the circumcision level was determined and recorded by the researcher according to the World Health Organization classification [2]. If the participant met the inclusion criteria into the study, then additional information was completed through a questionnaire.

Following the entry of each participant in labor, routine care in the delivery ward was performed by midwives, and the researcher played no roles in performing care, including decision making on cesarean section, induction, episiotomy, and even anthropometric (height, height, and head circumference at the time of birth) measurements and infant Apgar score. Finally, maternal outcomes were collected after delivery using patient records as well as direct observation. Maternal outcome included type of delivery (cesarean section or vaginal), duration of labor, episiotomy and perineal tears, need for oxytocin, postpartum hemorrhage, which were questioned and evaluated during the research steps. In addition, neonatal outcome included weight, height, and head circumference at birth, neonatal Apgar score, infant hospitalization, death at birth, maternal body mass index was recorded from the maternal and infant care booklet, mother’s height was measured by a meter. Labor duration was considered from the time when the client’s actual pains (regular contractions of less than 10 minutes and their intervals’ getting shorter) began. If the duration of the active labor (delivery) period is more than 8 hours in nulliparous women and the duration of the second stage of the labor is more than 2 hours in nulliparous women and the duration of the active labor step is more than 5 hours in multiparous women and the duration of the second stage of the delivery exceeds an hour, it was considered as a prolonged labor. In addition, the degree of perineal tears after delivery of the placenta was determined through observing and examining the perineal area using the World Health Organization classification by the researcher; First-degree tears that includes tears of the fourchette, perineal skin and vaginal mucosa and second-degree tears: In addition to rupture of the fourchette, perineal skin and vaginal mucosa, perineal muscles are also ruptured and third-degree tear in which the anal sphincter is ruptured as well in addition to the parts mentioned above and fourth-degree tears in which the rectal mucosa is torn and the rectal lumen is seen. The need for oxytocin was also determined based on use for augmentation or induction (labor induction).

Primary postpartum hemorrhage is the most common form of severe obstetric hemorrhages, defined as the loss of 500 ml or more of blood from the genital tract within the first 24 hours. Postnatal hemorrhage (bleeding) criterion were recorded based on the amount of bleeding according to the National Guide to Midwifery Services as follows; Mild: Less than 5 cm of blood-stained pad. Low: Less than 10 cm of blood-stained pad. Medium: less than 15 cm of blood-stained pad. Severe bleeding: If entire a pad is stained with blood within 2 hours and Very heavy bleeding: If the entire pad is stained with blood within 15 minutes or blood collects under the mother’s buttocks. The amount of bleeding was calculated and recorded by measuring the amount of impregnation of the pads, which were saturated with blood during the desired period immediately after delivery. The infant was weighed by a 100-g accuracy standard infant scale that had previously been weighed precisely. All babies were weighed by the same scale and the number of the weight observed was recorded by the delivery agent. In addition, height and head circumference was also measured by a meter and the Apgar score, which includes five criteria: appearance, pulse, reflex and irritability, activity, respiration. All the information were collected in the form of observation after the birth and were recorded by the delivery practitoners on the sheet of the infant file.After delivery, the delivery details and data on pregnancy outcomes of circumcised women as well as the uncircumcised ones were reported by the delivery practitoners were recorded on their case. After collecting the data, they were entered into statistical software spss16 and analyzed in spss16 to investigate the relationship between pregnancy outcome and circumcision.

Data were collected on demographic (including; maternal age, Place of Birth, Educational Stand status, and family Income), Current maternal outcomes (including; Gestational Age, parity, History of Abortion, History of Still Birth, Intended Pregnancy, Prenatal Care, No. of Prenatal Care, BMI, including Type of delivery, The first & second stage of labor duration, Episiotomy, Oxytocin administration for Induction and Augmentation, Type of Perinea the trauma, severity of Postpartum hemorrhage,) neonatal outcomes (Including Growth Parameters in Neonates and stillbirth and infant death, Infants hospital stay and appearance, pulse, grimace, activity, respiration (APGAR) scores). Circumcision and its level were confirmed using clinical examination according to the World Health Organization classification [2]. If the duration of the active labor (delivery) period is more than 8 hours in nulliparous women and the duration of the second stage of the labor is more than 2 hours in nulliparous women and the duration of the active labor step is more than 5 hours in multiparous women and the duration of the second stage of the delivery exceeds an hour, it was considered as prolonged labor. In addition, the degree of perineal tears after delivery was determined by examining the perineal area using the World Health Organization classification. The need for oxytocin was also determined based on use for augmentation or induction (labor induction). Primary postpartum hemorrhage is the most common form of severe obstetric hemorrhage defined as the loss of 500 ml or more of blood from the genital tract within the first 24 hours. All the information was collected in the form of observation after the birth and ordered by the delivery practitioners on the sheet of the infant file.

### Data analysis method

Mean and Standard deviation (SD) was performed to show the distribution of continuous variables and frequencies (%) were measured for qualitative variables. Categorical variables were analyzed using Chi-square (or Fischer’s exact tests). The independent sample t-test (or Mann–Whitney-U test) was used for analyses involving binary categorical subgroups. In order to parity was different in the two groups of circumcised and uncircumcised, but since parity is only related to pregnancy outcomes and it didn’t have a statistically significant association with the circumcision variable, there was no need for regression analyses. The data was recorded and analyzed with SPSS statistical software version 16 and *P*-value < 0.05 was considered significant respectively.

The study population consisted of 320 pregnant women, with 160 pregnant women in each group. Table 1 shows a comparison of Demographic characteristics and obstetric history in circumcised and uncircumcised patients. The mean age of circumcised women was significantly higher vs. Uncircumcised women (28.92(6.2) vs. 25.42(4.8); *P* < 0.001). The existence of circumcision was significantly higher in rural compared to urban areas (51.9% vs.18.1%; *P* < 0.001). The level of education of female levels parents, and spouse, Husband’s employment status were significantly associated with circumcision (P < 0.001). A significantly higher proportion of Uncircumcised women have intended pregnancy compared to circumcised women (94.4% versus 86.9%, *P* = 0.02). Parity of patients, and BMI were statistically different distributions between the two studied groups (*P* < 0.001, 0.003 respectively). 94.4% of uncircumcised nulliparous women and 86.9% of circumcised women experienced Intended Pregnancy (*P* = 0.02). Distribution of Income level, received Prenatal Care and no. of Prenatal Care, History of Abortion, and History of Still Birth were not statistically significant differences between the two groups.

**Table 1:** Comparison of Demographic characteristics and obstetric history in circumcised and uncircumcised patients

Variables		Circumcised Number (%)	Uncircumcised Number (%)	*P* Value
Place of Birth	City	77 (48.1)	131 (81.9)	< 0.001
	Village	83 (51.9)	29 (18.1)	
Educational Status	Illiterate	42(26.2)	7(4.4)	< 0.001
	Primary School	56(35)	52(32.5)	
	Guidance School/ High School	53(33.1)	83(51.9)	
	Academic Degree	9(5.6)	18(11.2)	
Income	Earnings > Expenses	40(25)	54(33.8)	0.1
	Earnings = Expenses	94(58.8)	90(56.2)	
	INCOME < EXPENSES	26(16/2)	16(10)	
Gestational Age, Weeks, Mean (SD)		39.5 (1.23)	39.3(1.14)	0.08
Parity	Nulliparous	48(30)	66(41.2)	< 0.001
	1	56(35)	68(42.5)	
	2≤	56(35)	26(16.3)	
History of Abortion		26(16.2)	24(15)	0.75
Of stillbirth		2(1.2)	2(1.2)	1
Intended Pregnancy		139(86.9)	151(94.4)	0.02
Prenatal Care		159(99.4)	156(97.5)	0.37
No. of Prenatal Care, Mean (SD)		6.1(2.25)	6.2)1.98)	0.54
BMI(Kg/M^2^)		27.42(5.1)	25.85(4.12)	0.003

Table 2 shows a Comparison of pregnancy outcomes in circumcised and uncircumcised patients. A significant difference in the duration of the second stage of labor was found between circumcised and uncircumcised women (*P* = 0.03). The Need for oxytocin in induction was significantly higher in circumcised mothers than in uncircumcised ones (*P* = 0.002). Perineal trauma II type (6.3% vs. 2.5%) and III type (3.1% vs. 2.5%) were significantly higher in circumcised than uncircumcised ones (*P* = 0.003).

**Table 2:** Comparison of pregnancy outcome in circumcised and uncircumcised patients

		Circumcised (%)	Uncircumcised (%)	P Value		
Type Of Delivery		140 (87.5)	139 (86.9)	0.87		
		20 (12.5)	21(13.1)			
Variables		236.96 (93.3)	231.55(120.96)	0.81		
Vaginal Delivery		19.22(2.44)	17.60 (23.76)	0.03		
Cesarean Section		94 (58.8)	100 (62.7)		0.42	
Oxytocin Administration	Induction	26(72.2)	7(5.4)	< 0.001		
	Augmentation	32(50)	10(32.3)	0.1		
Type of Perineal Trauma	I	41 (25.6)	23 (14.41)	0.003		
	II	10(6.3)	4(2.5)			
	II	5(3.1)	4(2.5)			
	IV	4(2.5)	0(0)			
	No tear	100(62.9)	129(80.6)			
Postpartum Hemorrhage	Mild	20(12.4)	24(15.1)	0.36		
	Low	55(34.2)	66(41.5)			
	Medium	81(50.03)	65(40.9)			
	Severe	3(1.9)	1(0.6)			
	Very severe	2(1.2)	3(1.9)			
Infant Death	Yes	1(0.6)	0	0.5		
Infant Hospitalization	Yes	12(7.5)	13(8.10)	0.83		

mode of delivery, Frequency of episiotomy, mean duration of the first stage of labor, Oxytocin administration for augmentation, neonatal hospital stay, and neonatal mortality incidence were not statistically significant differences between the two groups (*P* > 0.05).

Table 3 shows a Comparison of neonatal outcomes in circumcised and uncircumcised patients. Neonatal growth parameters were similar between the two groups. There was no statistically significant difference between Neonatal growth parameters including weight, height, and head circumference (*P* = 0.37, 0.21, 0.07 respectively). All the neonates in Uncircumcised and 97.5% of circumcised ones had an Apgar score of 8 to 10 respectively. An Apgar score of less than 3 was found in none of the studied neonates. None of neonates in uncircumcised women and 2.5% of the circumcised had Apgar score of e 4–7 and in Apgar score of neonates was not statistically significant difference between the two groups (*P* = 0.12).

**Table 3:** Comparison of neonatal outcomes in circumcised and uncircumcised patients

Variable		Circumcised (%)	Uncircumcised (%)	P Value
Birth Weight,gr, Mean (SD)		3357.06 (594.87)	3324.72 (437.54)	0.37
Height, Cm, Mean (SD)		49.42 (2. 55)	48.46 (4.64)	0.21
Head Circumference, Cm, Mean (SD)		34.21(1.98)	35.04 (1.98)	0.07
0–3		–	–	0.12
Apgar Scores 4–7		4(2.5)	0(0)	
8–10		156(97.5)	160(100)	

Table 4 shows the comparison of maternal outcomes in women based on I, II level circumcision. Level I circumcision was the most common type of circumcision among our patients (85.1%), and level II circumcision was seen in 14.9% of patients. Level III, IV circumcision was not observed among the studied population. We found no statistically significant difference in the duration of the first stage of labor based on I, II level circumcision (*P* = 0.53). A significant difference in the duration of the second stage of labor was found between women with level I circumcision compared women with level II circumcision (20.04 vs. 12.50 minutes, *P* = 0.03). 62.5%of women with level I circumcision and 37.5% compared women with level II circumcision experienced Episiotomy (*P* = 0.006).

**Table 4:** Comparison of maternal outcomes in women based on level I, and II circumcision

Outcomes		Level 1 (136)	Level 2 (24)	P Value
Postpartum Hemorrhage	**Mild**	24(17.6)	1(4.1)	0.21
	**Low**	56(41.2)	9(37.5)	
	**Medium**	53(39)	13(54.2)	
	**Severe And More**	3(2.2)	1(4.2)	
Type of Delivery	**Vaginal Delivery**	120(88.2)	20(83.3)	0.51
	**Cesarean Section**	16(11.82)	4(16.7)	
Episiotomy	**Yes**	85(62.5)	9(37.5)	0.02
Perineal Tear	**Yes**	83(61)	17(70.8)	0.36
The First Stage Of Labor, Minutes, Mean(SD)		238.24(94.86)	242.25(84.91)	0.53
The Second Stage Of Labor, Minutes, Mean (SD)		20.04(23.64)	12.50(9.93)	0.03

Table 5 shows the comparison of the outcome of pregnancy in the two studied groups based on maternal parity. A significant difference in the duration of the second stage of labor was found between circumcised and uncircumcised women who had a parity of 0 and 2 or more parity (P was 0.004 and 0.03, respectively). In nulliparous mothers, the Need for oxytocin was significantly higher in circumcised mothers than in uncircumcised ones (*P* value = 0.004). 16.7% of uncircumcised nulliparous women and 39.6% of circumcised nulliparous women experienced the tear (*P* = 0.006), and the corresponding rates were 15.9 and 60.4% for primipara women (*P* = 0.001).

**Table 5:** Comparison of the outcome pregnancy in the two studied groups based on maternal parity

Variable		Parity								
		0			1			2 and more		
		Uncircumcised Number (%)	Circumcised Number (%)	***P*** value	Uncircumcised Number (%)	Circumcised Number (%)	***P*** value	Uncircumcised Number (%)	Circumcised Number (%)	***P*** value
**Duration of the second stage of labor,minutes, mean(SD)**		29.61(23.7)	24.2(31.15)	0.007	15.15(14.13)	14.30(17.32)	0.23	15/25(25.7)	8.6(6.7)	0.03
**Episiotomy**				0.53			0.68			0.17
	**Yes**	46(69.7)	36(75)		50(72.5)	38(69.1)		5(20)	20(35.1)	
	**No**	20(30.3)	12(25)		19(27.5)	17(30.9)		20(80)	37(64.9)	
**Need to oxytocin**				0.004			0.09			0.09
	**Yes**	4(6.1)	12(25)		3(4.3)	7(12.7)		0(0)	7(12.3)	
	**No**	62(93.9)	36(75)		66(95.7)	48(87.3)		25(100)	50(87.7)	
**Rupture**				0.006			0.001			0.58
	**Yes**	11(16.7)	19(39.6)		11(15.9)	23(60.4)		9(36)	17(29.8)	
	**No**	55(83.3)	29(60.4)		58(84.1)	32(58.2)		16(64)	40(70.2)	

## Discussion

The current study aimed to evaluate the association of circumcision with neonatal and neonatal outcomes. The mean age of circumcised women was significantly higher compared with uncircumcised women. 80- 5 % of women were circumcision type I. The existence of circumcision was significantly higher in rural compared to urban areas. The level of female education, his parents, spouse, and husband’s employment status were significantly associated with circumcision. The higher mean duration of the second stage of labor, the Third and second degree of tear, and the need for oxytocin induction were significantly higher among circumcised women compared with uncircumcised women.

The present study shows that circumcised women are older than uncircumcised ones. The results of the present study are consistent with Ravansar’s results [8].

which indicated that the circumcision outbreak decreases. These findings are probably due to increasing education and enhancing health awareness and changing beliefs in recent years. In addition, females’ circumcision showed a significant relationship with the level of female education, parents, spouse, and husband’s employment status. This finding is consistent with Ethiopia and Egypt’s reports about this health problem [20, 21], which indicated the outbreak of females’ circumcision is associated with lower educational background among families. The present study showed that circumcision is more common in rural areas compared to urban areas; this result is consistent with a study conducted in Ethiopia where the majority of circumcised women lived in rural areas [20].

It seems that increasing the level of literacy will help to put an end to this harmful phenomenon. People empowerment and preventive policies are needed, and efforts should be made to end the inappropriate cultural and social norms that normalize and increase this practice. It seems Improving literacy could help to reduce this harmful phenomenon. Additionally, People empowerment and preventive policies should be considered.

Our results showed Circumcised women had higher body mass index (and number of deliveries, respectively Unintended pregnancy was more in circumcised women than in uncircumcised ones.

Our results showed the mean (SD) age of investigated circumcised women was 28.92 (6.2) years. The circumcision level in 85.1% of women was level 1 and 14.9% had level 2 circumcision. None of the participants had level III or IV circumcision. Agreed with these findings, Sudan [1], and Gambia [19, 22] researches shows level I and level II had the most common level of circumcision in these countries.

In contrast, in other research from metropolitan Sydney areas level III circumcision was the most type of Circumcision [3].

In our study, the mode of delivery was not associated with female circumcision, these findings are in line with other research by Chidebe et al. [23] and Varol et al., respectively [6].

However, review studies described increased cesarean delivery rates among circumcised women and this harmful phenomenon is definitely associated with poor neonatal care and increasing cesarean section rates [24, 25]. In contrast to the results of the present study, Frega et al.2013 [18], Balachandran et al. [12], Kiros et al. [26], and Sylla et al. [27] findings also showed a higher risk of cesarean section in circumcised women. In our study, multiparous women were included in the research and elective Cesarean-section cases were excluded, therefore Cesarean-section cases can probably depend on other causes of obstetrics and gynecology including higher frequency of level I&II circumcision, age of the studied population. Moreover, Levels of III&IV circumcision cause obstruction in the birth canal which is associated with higher cesarean section rates [27].

Kaplan et al. 2011 [19], Frega et al. 2013 [18], and Sylla et al. 2020 [27], studies indicate a longer duration of the second labor stage related to circumcision. Consistent with these studies, our results demonstrated that circumcision was a significant prognostic factor related to the higher mean duration of the second stage labor among the studied population.

However, Kiros et al. did not find a relationship between circumcision and the duration of the second stage of childbirth. Contrary to our observation, Kiros et al. [26] also found that level-one circumcised women had a higher mean duration of the second stage of labor [17]. Moreover, multiparous circumcised women were a longer duration of the second stage of labor than uncircumcised women, it can be due to elasticity reduction of the genital area and direct mechanical obstruction caused by all levels of circumcision that increased the duration of the second labor stage among circumcised women [17]. Moreover, the higher body mass index of circumcised women could be related to the higher duration of the second labor stage that resulted in dysfunctional or assisted delivery. In addition, the findings of the present study showed that being under episiotomy is similar in both groups of circumcised and uncircumcised women.

In our study, the use of episiotomy was similar in circumcised and uncircumcised women, and the Number of parity was not related to the use of episiotomy.

Despite these findings, the use of episiotomy in level-one circumcised women was higher than in level-two uncircumcised. Results from the current study are strongly supported by a meta-analysis study on 585 participants [17].

In contrast to these findings Akpak et al. 2020 [1] 2020, Berg et al. 2014, and Chidebe et al. 2019 [23] showed higher episiotomy rates among circumcised women.

The majority of circumcised women were level 1 and level 2 circumcision and we did not exclude multiparous women who do not need episiotomy, furthermore lower degree of circumcision was related to lower episiotomy rates. Results from the current study strongly support this recommendation.

In our study, a perineal tear was more common in circumcised women compared to uncircumcised ones and the and lower perineal tears were observed among patients with higher parity. Chidebe et al. [23], Kaplan et al. [19], Akpak et al. [1], Varol et al. [6] in 201 and Kiros Gebremicheal et al. 2018 showed female circumcision is associated with Perineal tear in delivery reported complications associated with the abnormal repair ruptures site is more common in circumcised women. It seems Circumcision has adverse effects on the condition and tightness of perineal and vaginal tissue that causes perineal tear.

This study demonstrates that postpartum hemorrhage is not related to Circumcision. This result in line with the research by Chidebe et al. who did not confirm a relationship between bleeding and circumcision [23].

In contrast with our finding, a review of the literature demonstrates that circumcision increases the risk of bleeding from the episiotomy area, perineal tear, and postpartum hemorrhage among circumcised women [13, 24–26, 28]. It could be related to the Variation in female circumcision level in our studied population.

In our studied population, female circumcision increases the risk of oxytocin administration for induction. These findings are in line with the research by Frega et al. [18] 2013 which revealed Oxytocin administration for induction in the second labor stage is higher in circumcised women than in uncircumcised ones. Time by increasing uterine contractions [18].

in the most cases spontaneous uterine contractions plus voluntary abdominal pressure are insufficient [24]. therefore, Oxytocin is administered in during the second labor stage to reduce the expulsion time by increasing uterine contractions [18].

In this study, we compared neonatal outcomes between two groups. In studied neonates, An Apgar score of less than 3 was found none of studied neonates. Neonatal growth parameters, Apgar score, and neonatal death frequency were not statistically significant differences between the two groups.

In line with these findings, Odoru et al. 2006 [29] and Akpak et al. 2020 [1] report that circumcised women neonates had similar outcomes compared to uncircumcised women neonates. An earlier systematic review and meta-analysis study supported the similarity of the neonates’ Apgar score between circumcised and uncircumcised women [28]. In contrast with our results, Rashid Soleiman et al. 2021 [30] and Frega et al. 2013 [18] showed that circumcision is a prognostic factor low Apgar score of 5 minutes.

The association between higher levels of maternal circumcision and neonatal outcomes (including; stillbirth, need to ICU, extended hospitalization stay, and decrease in Apgar score) was shown in reports from African countries [13, 31, 32], but these associations were not supported by European countries reports [12, 33, 34] these findings highlight the effect of other important factors in the delivery process including proper hospital conditions, proficient staff, and proper management of the delivery process.

Contrary to the results of the present study, a number of researchers have identified female genital circumcision as an independent risk factor for increased maternal and fetal mortality. Additionally, Kaplan et al. 2013 showed higher practical interventions for intrauterine resuscitation of a distressed fetus among level-2 and 3 circumcised mothers compared to uncircumcised ones [19]. Additionally, Obermeyer in 2005 reported circumcision does not increase maternal and neonatal mortality but increases the risk of some pregnancy and delivery complications [35]. WHO study group reports first- and second-level circumcision is associated with higher infant mortality [13]. Reduced perineal tissue elasticity and obstruction following female circumcision (infibulation) can prolong the second labor stage that is contributing to the increased rate of fetal death, perinatal tear, and perineal episiotomy.

## Conclusion

The present study showed that circumcision was associated with the mean duration of the second stage of labor, need for oxytocin in induction, and the frequency of degree-2 and degree 3 tear in circumcised women. These findings highlight that circumcised women need intensive care during delivery and may further help formulate obstetric and gynecological care policies. More studies on the psychological and mental consequences and sexual problems of the affected women were warranted.

## Data Availability

The datasets generated and/or analysed during the current study are not publicly available due to limitations of ethical approval involving the patient data and anonymity but are available from the corresponding author on reasonable request.
